# Molecular Connections between Cancer Cell Metabolism and the Tumor Microenvironment

**DOI:** 10.3390/ijms160511055

**Published:** 2015-05-15

**Authors:** Calvin R. Justus, Edward J. Sanderlin, Li V. Yang

**Affiliations:** 1 Department of Internal Medicine, Brody School of Medicine, East Carolina University, Greenville, NC 27834, USA; E-Mails: justusc11@students.ecu.edu (C.R.J.); sanderline09@students.ecu.edu (E.J.S.); 2 Department of Oncology, Brody School of Medicine, East Carolina University, Greenville, NC 27834, USA; 3 Department of Anatomy and Cell Biology, Brody School of Medicine, East Carolina University, Greenville, NC 27834, USA; 4 Lineberger Comprehensive Cancer Center, University of North Carolina, Chapel Hill, NC 27599, USA

**Keywords:** tumor microenvironment, cancer cell metabolism, hypoxia, acidosis, cancer therapy

## Abstract

Cancer cells preferentially utilize glycolysis, instead of oxidative phosphorylation, for metabolism even in the presence of oxygen. This phenomenon of aerobic glycolysis, referred to as the “Warburg effect”, commonly exists in a variety of tumors. Recent studies further demonstrate that both genetic factors such as oncogenes and tumor suppressors and microenvironmental factors such as spatial hypoxia and acidosis can regulate the glycolytic metabolism of cancer cells. Reciprocally, altered cancer cell metabolism can modulate the tumor microenvironment which plays important roles in cancer cell somatic evolution, metastasis, and therapeutic response. In this article, we review the progression of current understandings on the molecular interaction between cancer cell metabolism and the tumor microenvironment. In addition, we discuss the implications of these interactions in cancer therapy and chemoprevention.

## 1. Introduction

In the early twentieth century Otto Warburg pioneered the work that investigated a metabolic phenomenon found in the majority of cancers, which is now known as the “Warburg effect” [1,2,3,4]. Warburg originally hypothesized that mitochondrial impairments that lead to irreversibly defective respiration of cells could cause the development of cancer [3,4]. This hypothesis was driven by the observation that cancer cells exhibited an increased glycolytic phenotype in comparison to untransformed cells even in the presence of oxygen [2,4]. However, the idea of respiratory impairment in the development of cancer has shifted and changed over time as respiration is now known to remain relatively active in many types of tumor cells when oxygen is present [5,6,7]. However, the observation that tumor cells exhibit a higher rate of glycolysis still holds true for most cancers.

**Figure 1:**
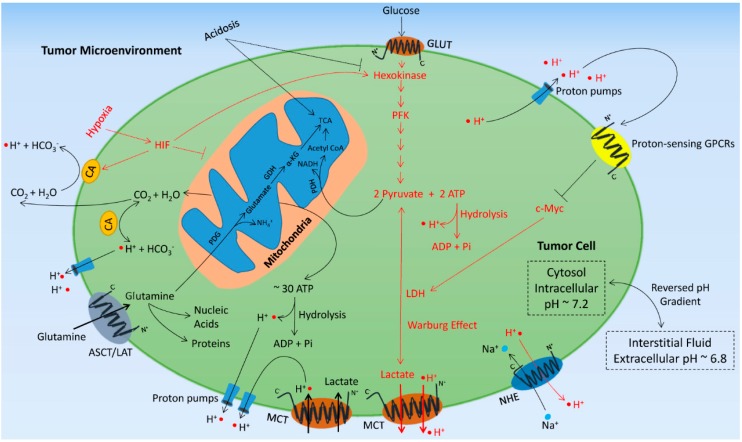
The complex interactions between cancer cell metabolism and the tumor microenvironment. Cancer cells exhibit increased glycolysis even in the presence of oxygen (Warburg effect) and under hypoxic conditions glycolysis may be further stimulated (shown in red). The stimulation of glycolysis increases proton production and facilitates proton efflux via an array of acid transporters such as MCT, NHE, and proton pumps, causing acidosis in the tumor microenvironment. Acidosis acts as a negative feedback signal by lessening glycolytic flux and facilitating mitochondrial respiration (shown in black). ASCT: Na^+^-dependent glutamine transporter; CA: carbonic anhydrase; GDH: glutamate dehydrogenase; GLUT: glucose transporter; GPCR: G-protein-coupled receptor; HIF: hypoxia inducible factor; LAT: Na^+^-independent glutamine transporter; LDH: lactate dehydrogenase; MCT: monocarboxylate transporter; NHE: sodium/hydrogen exchanger; PDG: phosphate-dependent glutaminase; PDH: pyruvate dehydrogenase; PFK: phosphofructokinase; TCA: tricarboxylic acid cycle.

There have been recent investigations that have shed light into how the “Warburg effect” may occur. It is becoming evident that both genetic and environmental factors contribute to the “Warburg effect” observed in tumor cells. It was found that certain oncogenes and tumor suppressors can directly regulate tumor cell metabolism. Seminal studies on the *c-Myc* oncogene demonstrated that c-Myc can increase the expression of genes involved in glycolysis, such as lactate dehydrogenase-A (*LDH-A*) and glucose transporter 1 (*GLUT1*), and stimulate glycolytic metabolism of cancer cells [8,9]. More recent studies revealed that tumor protein p53 (TP53), a key tumor suppressor frequently mutated in cancers, inhibits glycolysis and increases mitochondrial respiration in cancer cells [10]. It is now widely recognized that besides their traditional roles in controlling cell cycle and apoptosis many oncogenes and tumor suppressors can also regulate cell metabolism [11]. In addition to the genetic factors, the “Warburg effect” is also regulated by environmental factors found in the tumor. As a result of defective vascularization and reduced blood perfusion, regions of the tumor microenvironment are hypoxic and acidic. Hypoxia and acidosis have complex effects on cell metabolism as well as cancer cell somatic evolution, oncogene and tumor suppressor function, metastasis, and therapeutic response to chemotherapeutics [12,13,14,15]. It is well established that hypoxia, mainly mediated through the hypoxia-inducible factors (HIFs), enhances the “Warburg effect” by up-regulating glycolytic genes such as *hexokinases*, *LDH-A*, and *GLUT* [16]. In contrast, acidosis has recently been shown to suppress glycolysis and augment mitochondrial respiration in cancer cells [17,18]. These observations illustrate the close and complex interaction between cancer cell metabolism and the tumor microenvironment (Figure 1).

In this review we will describe how cancer cell metabolism may shape and modify the tumor microenvironment. In addition, we will detail the current understanding for how two specific environmental factors present in the tumor microenvironment, hypoxia and acidosis, reciprocally affect cancer cell metabolism. Lastly, we will discuss how molecular signaling pathways associated with metabolic alterations in cancer cells as well as hypoxia and acidosis in the tumor microenvironment can be exploited to develop new approaches for cancer therapy and prevention.

## 2. Hypoxia Is a Hallmark of the Tumor Microenvironment

Hypoxia is the low oxygen concentration within solid tumors as a result of abnormal blood vessel formation, defective blood perfusion, and unlimited cancer cell proliferation. As tumor growth outpaces that of adequate vasculature, oxygen and nutrient delivery become insufficient. This dynamic interplay between the normal stroma and the malignant parenchyma, coupled with inevitable hypoxia, is common in any solid tumor microenvironment. The progression of hypoxia over time is a consequence of increased oxygen consumption by abnormally proliferating cancer cells, which also produce an acidic environment. In this sense unlimited tumor cell proliferation is a cancer hallmark interrelated with hypoxia and acidosis. Hypoxia facilitates a preferentially up-regulated glycolytic phenotype for necessary biosynthetic intermediates and oxygen independent ATP production. At first, the glycolytic phenotype seems like an inefficient means of energy production for the cancer cell [1]. Glycolysis generates two lactic acid and two ATP molecules from each glucose molecule. Comparatively, oxidative phosphorylation generates about 30 molecules of ATP from each glucose molecule. In terms of energy efficiency, tumor cells should rely less on glycolysis and preferentially utilize oxidative phosphorylation. However, this is not the case. The glycolytic phenotype, nonetheless, is a necessary and critical step for tumor cells to adapt and survive under hypoxic stress. This adaptation is a heritable conversion and reoccurs in non-hypoxic regions of the tumor. In addition, increased glycolysis acidifies the extracellular environment causing apoptosis for cells, such as neighboring stromal cells that are not capable of survival in this extreme environment.

Tumor development is tightly regulated by the growth of vasculature. Increased vasculature facilitates the delivery of nutrients and removal of toxic byproducts to further cell growth [19]. Tumors maintain slow growth and/or dormancy when they are 1–3 mm^3^ in size due to an avascular phenotype [20]. Cellular proliferation is suggested to balance with apoptosis in this avascular stage maintaining the reduced tumor size [21]. When tumor cells upregulate excretion of pro-angiogenic factors, the “angiogenic switch” occurs where the promotion of new vascularization increases blood flow, nutrient deposition, and subsequent tumor growth [22]. This switch is due to the counterbalancing of angiogenic inducers over inhibitors. In angiogenesis, tumor associated endothelial cells (TECs) are common stromal cells that sprout from pre-existing blood vessels resulting in angiogenesis [23]. The blood vessel formation pattern found in the tumor microenvironment is highly irregular in size, shape, branching, and organization [24,25]. The blood vessel function is also inadequate. This phenomenon is likely mediated by the hypoxic regions of the tumor where pro-angiogenic growth factors are persistently produced, causing continuous vasculature remodeling [26]. The TECs do not bind to each other as tightly as normal blood vessels, leading to leaky blood vessels that allow hemorrhaging and plasma leaks [27]. The characteristic leakiness of these blood vessels is in some measure due to abnormalities in pericyte coverage and function [28]. Pericytes on TECs are loosely attached and have abnormal morphology leading to less stable EC interactions [28,29]. This contributes to the poor blood perfusion and inadequate delivery of nutrients and oxygen to the tumor coupled with an increased ability of metastasis and intravasation by tumor cells [30]. Vascular endothelial growth factor (VEGF) is a major pro-angiogenic factor that is regulated by the hypoxia inducible factor 1 alpha (HIF-1α) [31]. HIF-1 is stabilized by low oxygen availability and is translocated to the nucleus to regulate the expression of numerous genes including *VEGF*. This indicates the presence of a feedback mechanism whereby nutrient and oxygen deprived tumor regions promote angiogenesis [32].

## 3. Molecular Connections between Hypoxia and Cancer Cell Metabolism

Under hypoxia, cancer cells must adapt in order to maintain tumor growth. The environmental stress of hypoxia triggers molecular changes that facilitate metabolic adaptations. Some such alterations are up-regulated glycolytic enzyme expression coupled with oxidative phosphorylation inhibition, mitochondrial selective autophagy, and glucose-independent citrate production for fatty acid synthesis. Hypoxia-inducible factor (HIF) activation is the most understood and characterized molecular response governing many of these altered metabolic pathways of hypoxia [33]. There are three isoforms of HIF of which include HIF-1, HIF-2, and HIF-3 [34]. The HIF-1 and HIF-2 isoforms have the closest homology, yet discrete molecular targets [35,36]. HIF-1 is the most characterized of all isoforms in the molecular response to hypoxia in upregulating glucose metabolism to promote cell survival [37]. One study suggests HIF-2 does not primarily regulate glucose metabolism as HIF-1, but rather regulates cell cycle progression through the interaction with oncoprotein c-Myc [37,38]. In addition to HIF signaling, there are some HIF independent, O_2_ sensing, hypoxic responses such as the mammalian target of rapamycin (mTOR) inhibition of which will be discussed.

The HIF responds to low oxygen concentrations within the hypoxic tumor microenvironment [33]. HIF-1 is a heterodimeric transcription factor that is composed of an α and β subunit. The α subunit is oxygen sensitive and the β subunit is constitutively stable [33]. The von Hippel-Lindau protein (pVHL) is a tumor suppressor that is involved in the regulation of HIF-1. Many studies have demonstrated the role of VHL in clear cell renal carcinoma where the *VHL* gene can be inactivated by mutations, leading to stabilization of HIF-1 and its subsequent transcription of target genes [39]. The stability of the HIF-1α subunit is oxygen dependent [40]. Under normoxic conditions, the prolyl hydroxylase domain (PHD) enzymes hydroxylate two proline residues (Pro402/Pro564) on the oxygen dependent degradation domain (ODDD) of HIF, which initiate the HIF-1α subunit interaction with the ubiquitin E3 ligase complex within the VHL tumor suppressor complex [41]. The subsequent ubiquitination of HIF-1α initiates degradation via the proteasome. Conversely, under low oxygen concentration, VHL is unable to bind to HIF α and HIF is thereby stabilized and bypasses degradation. HIF-1α is then translocated into the cell nucleus to regulate the transcription of its target genes.

It has been well observed that cancer cells preferentially up-regulate glycolysis in response to HIF-1 activity [42,43,44,45]. HIF-1 positively regulates the transcription of over 100 genes, of which many directly up-regulate glycolysis [46]. To increase glycolytic flux, glucose transporters GLUT1 and GLUT3 expression is increased by HIF-1. This increases the availability of glucose within the cytoplasm for energy production. HIF-1 then facilitates the conversion of glucose to pyruvate by increasing the expression of glycolytic enzymes such as hexokinase 1/2 (HK I/II) and pyruvate kinase M2 (PKM2). Not only does HIF-1 activation increase glycolysis, but also directly inhibits oxidative phosphorylation by blocking pyruvate entrance into the TCA cycle [47]. HIF-1 accomplishes this by reducing mitochondrial biomass and up-regulating genes that directly inhibit oxidative phosphorylation. Two HIF-1 target genes are pyruvate dehydrogenase kinase 1 (*PDK1*) and *LDH-A* [47,48]. PDK1 inactivates pyruvate dehydrogenase (PDH) to inhibit the conversion of pyruvate into acetyl CoA. Therefore, PDK inhibits flux through the TCA cycle and may increase pyruvate availability for conversion into lactate by LDH-A [47]. Furthermore, HIF-1 activates *PKM2* gene transcription [49]. In turn PKM2 enhances HIF-1 binding to the hypoxia response element (HRE) and the recruitment of the p300 co-activator [50,51]. This creates a positive feedback loop whereby PKM2 promotes HIF-1 transactivation [51]. HIF-1 also positively targets BCL2/adenovirus E1B 19 kd-interacting protein 3 (*BNIP3*) expression under hypoxic stress [49]. This leads to a reduction in mitochondrial activity and prevents reactive oxygen species (ROS) generation that is produced from oxidative phosphorylation. ROS production from complex III of the electron transport chain in the mitochondria has been shown to stabilize and thereby promote HIF-1 activity [52]. This is most likely done by the oxidation of Fe^2+^ to Fe^3+^ and inactivating PHD activity for the hydroxylation of the ODDD on HIF-1 [53]. Attenuation of oxidative phosphorylation and increased mitochondrial selective autophagy reduces harmful, yet characteristic ROS production in the tumor microenvironment. This presents a possible feedback loop whereby subsequent HIF stabilization by ROS increases PDK1 and BNIP1 expression to prevent further ROS production and maintain the glycolytic phenotype of tumor cells. It has further been suggested that this potential feedback loop keeps tumor cells out of senescence and initiates increased vascularization by HIF-1 [54,55]. Additionally, hypoxia has been shown to initiate macroautophagy by the activation of AMPK, a major regulator in energy homeostasis, independent of HIF-1, BNIP3, or BNIP3L [56]. These data suggest that the induction of autophagy is a protective mechanism activated in response to hypoxic stress [56]. Furthermore, HIF activation has been shown to regulate cytochrome oxidase COX-4 subunit composition, which further demonstrates the ability of HIF-1 to prevent harmful ROS production under various oxygen concentrations [57]. The regulation of HIF-1α and mitochondrial ROS under hypoxia is multifarious and a clear conclusion has yet to arise [58,59,60].

HIF-1α protein and mRNA levels are not only regulated by O_2_ availability, but also hormones and growth factors [61]. One such example of HIF-1α regulation is the stimulation of the PI3K/Akt pathway by insulin, of which can ultimately regulate HIF-1α by glycogen synthase kinase 3 (GSK-3) by directly phosphorylating HIF-1α [62]. Activation of GSK-3 has shown to inhibit HIF-1α activity by reducing HIF-1α protein levels [63]. Another study demonstrated that the activation of Akt/PKB in hepatocellular carcinoma cells by insulin can inhibit GSK-3 activity and thereby prevent HIF-1α degradation. This observation provides a mechanism whereby HIF-1α can be regulated in a VHL-independent manner [62].

Hypoxic tumor cells require the necessary building blocks for survival and proliferation. Fatty acids are required for cellular membrane biosynthesis and signaling during proliferation. It is known that fatty acid biosynthesis is stimulated in cancer cells under hypoxic stress [64,65]. HIF-1 is both directly and indirectly involved in lipid metabolism. HIF-1 stabilization inhibits mitochondrial oxidative phosphorylation and thereby inhibits fatty acid synthesis from glucose-sourced carbon by shunting pyruvate away from the mitochondria. Hypoxic cells must use an alternative carbon source for *de novo* fatty acid synthesis. It has been shown that hypoxic cells preferentially depend on reductive carboxylation of glutamine derived α-ketoglutarate (α-KG) by reversing the NADPH-linked mitochondrial isocitrate dehydrogenase (IDH2) and aconitase reactions in the TCA cycle. Cytoplasmic IDH1 is then involved to generate cytosolic citrate for lipid synthesis [66,67]. This process can occur under normoxic conditions in certain cancer cell lines as reductive carboxylation of glutamine derived carbon is observed in VHL deficient renal cell carcinoma cell lines [66,67]. With the glutamine-derived cytosolic acetyl CoA, HIF-1 and protein kinase B (AKT) activation has been shown to up-regulate fatty acid synthase (FAS) for acetyl CoA to fatty acid conversion. Hypoxia has been demonstrated to enhance fatty acid synthesis via the sterol regulatory-element binding protein (SREBP)-1, which is a transcriptional regulator for the *FAS* gene, in multiple types of cancer [68,69]. SREBP-1 induction follows activation of HIF-1 by the phosphorylation of AKT [69]. The enhanced lipogenesis allows the hypoxic cells to store triglycerides in lipid droplets. Recent studies show that HIF-1α is a predominate factor in lipin1 expression by binding to a distal HRE in the *lipin1* gene promoter [70].

Cancer cell metabolism is not solely regulated by HIF under hypoxia; there are a number of HIF-independent mechanisms. One such mechanism is that of mTOR regulation. Cellular growth and proliferation is handicapped without mRNA translation. This process, however, requires a high-energy investment by the cell. Under hypoxic stress, the cancer cell will adapt for survival by limiting the costly energy investment for mRNA translation through preventing mRNA translation at the initiation stage in responses to environmental nutrient stressors [71,72,73]. mTOR regulates cellular survival and growth by means of modulating mRNA translation, ribosomal biogenesis, metabolism, and autophagy from the phosphorylation of S6K, 4E-BP1, and eEF2K [74,75]. mTOR has profound impacts on cellular metabolism. mTOR is the downstream effector of the RTK-PI3K-AKT-mTOR signaling cascade. In various types of cancer, this signaling pathway is altered. There are many mechanisms by which mTOR regulation is mediated; however, one study shows that under energy starvation, which is associated with hypoxia, mTOR can be regulated through the AMPK/TSC2/Rheb pathway, independent of HIF-1 [76]. Hypoxia and energy depletion regulates cellular metabolism by inhibiting target of rapamycin kinase complex 1 (TORC1) activity. The tuberous sclerosis tumor suppressors (TSC1/2) and the hypoxia inducible gene *REDD1* are essential to effectively regulate TORC1 activity by hypoxia. Studies have shown that REDD1 suppresses mTORC1 activity by the disassociation of TSC2 from inhibitory 14-3-3 proteins [77].

## 4. Acidosis Is a Hallmark of the Tumor Microenvironment

Acidosis is another characteristic feature of the tumor microenvironment [13,78,79,80,81]. Normal tissue pH in humans is tightly regulated around pH 7.4. The pH of the tumor microenvironment, however, may range from pH 5.5 to 7.4 [13,78,79,80,81]. The tumor microenvironment is in constant flux. Solid tumors can become regionally acidic for varying periods of time [82,83]. The development of acidosis in the tumor microenvironment is dependent on blood perfusion and cancer cell glycolytic metabolism [13,78,84]. Reduced blood perfusion increases anaerobic metabolism resulting in lactic acid production. Even in the presence of oxygen, cancer cells preferentially use glycolysis for energy production generating a large amount of lactic acid. Other sources of protons in the tumor microenvironment are derived from the hydrolysis of ATP as well as hydration of carbon dioxide (CO_2_) by carbonic anhydrases, among others [85,86]. Over time lactate and protons are exported from cancer cells by monocarboxylate transporters, vacuolar type H^+^-ATPases, Na^+^/H^+^ exchangers, and other acid-base transporters [87,88,89]. Due to defective blood perfusion and inefficient removal, protons and lactate accumulate in the tumor interstitial space. The export of protons increases the survival and proliferation of tumor cells by alkalizing the intracellular pH and causes a reversed pH gradient by acidifying the extracellular tumor microenvironment [78,90,91,92,93].

The tumor microenvironment is a complex milieu of cancer cells, stromal cells, infiltrating immune cells, and blood vessels. Thus understanding the diverse cellular responses to a common characteristic in the tumor microenvironment such as extracellular acidosis is important for a more complete view of tumor biology. As the tumor microenvironment is in constant acidotic flux it is becoming evident that the effects of acidosis on tumor cell biology should be viewed in terms of acute *versus* chronic [14]. Acute acidosis may decrease cancer cell proliferation, stimulate apoptosis, and cause cell cycle arrest [94,95,96]. In contrast, chronic acidosis is a Darwinian selection pressure that induces somatic evolution of cancer cells by modifying genomic stability through gene mutations, clastogenicity, and chromosomal instability [97,98,99]. Within the tumor microenvironment, acquiring multiple genomic mutations over time may provide benefits for cancer cell adaptation. Chronic exposure to acidosis can select for acidosis resistant cancer cells that may regain high proliferation rates. In addition to cancer cells, the surrounding stromal cell response to acidosis adds to the complexity of tumor biology [14]. In immune cells, such as in neutrophils, acidosis may stimulate their activity [100]. In other cells, such as natural killer cells and CD8^+^ T lymphocytes, acidosis may be functionally repressive [101,102]. In addition, immune cell presence may further reduce tumor pH through respiratory bursts or by increasing oxygen consumption [103,104]. Acidosis has also been shown to modulate angiogenesis and vascular endothelial cell inflammation [105,106,107,108]. In summary, acidosis in the tumor microenvironment has multiple effects on cancer cells and stromal cells in a tumor.

## 5. Molecular Connections between Acidosis and Cancer Cell Metabolism

As some of the molecular networking between hypoxia and cancer cell metabolism are introduced in the previous sections this section will be detailing the molecular connections between acidosis and tumor cell metabolism. The tumor cell response to acidosis is complex and is dependent on cell type as well as oxygen and nutrient availability. Whereas hypoxia and acidosis induce distinct biological effects, the tumor cell response to both stimuli simultaneously can have synergistic or antagonistic effects on certain cellular processes. Simultaneous treatment of MCF7 and SUM52PE breast cancer cells with acidosis and hypoxia may induce the expression of inflammatory response genes such as tumor necrosis factor alpha (*TNF-α*) and tumor necrosis factor alpha-induced protein 3 (*TNFAIP3*) [109]. Several genes that are linked to the unfolded protein response such as C/EBP homology protein (*CHOP*), x-box binding protein-1 (*XBP-1*), activating transcription factor-3 (*ATF3*), activating transcription factor-4 (*ATF4*), and eukaryotic translation initiation factor 2-alpha (*eIF2α*) are also highly induced in MCF7 and SUM52PE breast cancer cells when co-treated with acidosis and hypoxia [109]. Increased expression of ATF4 was found as advantageous for MCF7 and SUM52PE breast cancer cell survival while recovering from acidosis and hypoxia treatment [109]. ATF4 may also play a role in tumor cell survival to endoplasmic reticulum (ER) stress and severe hypoxia by driving the expression of unc-51 like autophagy activating kinase 1 (ULK1), a regulator of autophagy in A431 and MCF7 cells [110]. On some other aspects, however, acidosis and hypoxia may antagonize each other. Treatment with acidosis under hypoxic conditions antagonizes the expression of a specific subset of hypoxia related genes such as carbonic anhydrase 9 (*CA9*), phosphoglycerate kinase 1 (*PGK1*), and stanniocalcin 1 (*STC1*) [109]. The down-regulation of hypoxia induced genes by acidosis was proposed to occur through inhibiting the translation of HIF1-α [109]. In prostate cancer cells, acidosis lessens glycolytic activity by reducing the expression of LDH, PFK, and fructose-1, 6 bisphosphatase via reduced AKT activity whereas hypoxia increased glycolysis [17].

There are several molecular mechanisms whereby acidosis may alter tumor cell metabolism. p53 is an important regulator of the metabolic response to acidosis. The ability of acidosis to activate p53 and stimulate the TCA cycle through inhibition of glycolysis has been demonstrated. For example, acidosis induced p53 expression may transcriptionally inhibit the expression of glucose transporters GLUT1 and GLUT4 in specific tissues, thereby effectively reducing glucose availability for glycolysis [111]. In addition, acidosis is reported to activate p53 and increase expression of glucose 6 phosphate dehydrogenase (G6PD) and glutaminase 2 [112]. This is suggested to direct glucose towards the pentose phosphate pathway (PPP) as well as increase glutaminolysis [112]. This may also drive the TCA cycle through the production of metabolic intermediates and increase the amount of NADPH in the cell to counteract ROS production. p53 activation may also induce the expression of Parkin (*PARK2*), a Parkinson disease-associated gene, to reduce glycolytic activity [113]. PARK2 regulates the expression of pyruvate dehydrogenase alpha 1 (PDHA1), a critical component for the activity of pyruvate dehydrogenase (PDH) [113]. PDHA1 knockdown increases glucose uptake, rate of glycolysis, and lactate production, facilitating the “Warburg effect” [113]. This gives PARK2 the ability to effectively reverse the “Warburg effect” by inducing PDHA1. Moreover, PARK2 also regulates expression of reduced glutathione (GSH), a major antioxidant and ROS scavenger in the cell [113]. This is proposed to occur through activation of p53 and may reduce ROS when oxidative phosphorylation is increased [113]. Furthermore, γ-irradiation-induced tumorigenesis is sensitized following the knockout of PARK2 in C57BL/6J mice, indicating the *PARK2* gene as a tumor suppressor [113]. The ability for p53 to regulate cancer cell metabolism by reducing glycolysis and increasing oxidative phosphorylation while simultaneously mitigating ROS is crucial for understanding acidosis induced metabolic alterations in the tumor.

p53 also plays an important role in acidosis induced cell death. In some cell types acidosis stimulated cell death has been deemed a p53 dependent response [94,95]. In addition, bypassing chronic acidosis related cell death in many tumor cells has been correlated with p53 loss-of-function mutations [95]. This is an intriguing phenomenon as many cancer types have p53 mutations. However, acidosis induced cell death is not always dependent on p53 function. Tumor cells may bypass acidosis stimulated cell death through the autophagy response. As previously mentioned acidosis induces mutations in nuclear DNA leading to chromosomal instability and clastogenicity [97,98,99]. Intracellular acidosis may also induce mutations in mitochondria DNA and may damage organelles. In response to acidosis induced organelle damage and altered energy status, autophagy permits the recycling of old or damaged organelles. Chronic acidosis treatment in a variety of cell types such as MDA-MB-231 and HS766T breast cancer cells and Me30966, Mel501, WM793 melanoma cells has been found to stimulate autophagy [114,115]. Furthermore, *in vitro* treatment with acidosis increases the expression levels of key regulators of autophagy such as autophagy related 5 (ATG5) and BNIP3 while increasing the number of autophagic vacuoles [115]. Other regulators of autophagic vacuole formation such as microtubule-associated protein light chain 3 (LC3) are also up-regulated following subcutaneous injection of MDA-MB-231 cells into nu/nu mice [115]. *In vivo*, buffering MDA-MB-231 tumors with sodium bicarbonate (NaHCO_3_) reduced the expression of LC3, indicating autophagic vacuole formation is due to tumor acidosis [115]. p53 has been investigated as a key regulator of autophagy. p53 can activate autophagy through transcription independent mechanisms, such as AMP-activated protein kinase (AMPK) and mTOR [116,117]. Conversely, p53 can activate autophagy through transcription dependent mechanisms, such as damage-regulated autophagy modulator (DRAM), phosphatase and tensin homolog (PTEN), and tuberous sclerosis 1 (TSC1) [116,117]. Interestingly, AMPK is also activated following chronic treatment with acidosis as a result of abrogated ATP levels [114]. AMPK is an important energy sensor in the cell. It is activated in response to energy stress, e.g., hypoxia and nutrient deprivation, in order to transduce several signals that inhibit anabolic metabolism and activate catabolic processes [118]. AMPK activation can induce the activity of TSC1/TSC2 and subsequently reduce mTOR activity thereby stimulating autophagy. AMPK increases the activity of forkhead box O3 (FOXO3), p53, cyclin-dependent kinase inhibitor 1B (p27), TSC2, and Raptor in response to cellular stressors like acidosis, hypoxia, and nutrient deprivation [118]. AMPK may also increase apoptosis, induce autophagy, and reduce cell growth of which all reduce the likelihood of cancer development [119]. During chronic selection, however, autophagy has been described as a mechanism for cancer cells to resist cell death from stressors such as chemotherapy, hypoxia, or acidosis [114,115,120,121]. In this way autophagy may reduce cell death and allow for the selection of acidosis, hypoxia, or chemotherapy resistant cells leading to advanced tumor development and malignancy.

The effects of acidosis on oncogene expression have been briefly investigated. In some cell types acidic pH treatment may reduce the expression of several proto-oncogenes and inhibit oncogenic pathways. In melanoma cells chronic acidosis reportedly reduced the expression of mTOR through the activation of AMPK [114]. Interestingly, AKT, of which mTOR is a downstream effector, is reportedly reduced following acidosis treatment in prostate cancer cells [17]. The *AKT* proto-oncogene is widely over-expressed in a variety of cancers. It is an important activator of cell proliferation, cell survival, glycolysis, cell motility, and angiogenesis of which all are essential for tumor growth. AKT also has been found to phosphorylate Na^+^/H^+^ transporter NHE-1 at residue Ser648 inhibiting its activity during intracellular acidosis [122,123]. Reduced AKT activity in response to acidosis may encourage increased activity of NHE-1 and subsequently increase proton export, permitting for intracellular alkalization and cell proliferation [122,123]. In lymphomas, acidosis has been demonstrated to reduce c-Myc expression [124]. *c-Myc* is a proto-oncogene that drives the transformation of many lymphomas as well as other cancer types [125,126]. The activity of c-Myc may also drive the expression of proteins that promote glycolysis and tumor acidity of which include LDH-A and GLUT1 as well as enzymes that promote nucleotide synthesis and ATP production [8,9,127,128]. However, in other cell types such as neuroblastoma and H1299 lung cancer cells, acidosis stimulates the expression of oncogenes such as *c-Myc* and high mobility group box 1 (*HMGB-1*) [124,129]. Acidosis may also stimulate epigenetic rearrangements to increase the expression of a novel metabolic proto-oncogene fatty acid synthase (*FAS*) in breast cancer cells [130]. The increased expression of FAS is possibly due to the need for increased amounts of lipids to provide dividing cells with the materials to build cell membranes.

Extracellular acidosis may induce a wide variety of effects in tumor cell function and metabolism by stimulating cell signaling. Molecular mechanisms by which acidosis may directly regulate these signaling pathways, however, are poorly understood. Acidosis activates a family of proton sensing G-protein coupled receptors, including G-protein coupled receptor 4 (GPR4), G-protein coupled receptor 65 (GPR65), G-protein coupled receptor 68 (GPR68), and G-protein coupled receptor 132 (GPR132) [14,131,132]. GPR68 and GPR4 have been reported as tumor metastasis suppressors [133,134]. The overexpression of GPR68 in PC3 prostate cancer cells reduced metastasis to the stomach, diaphragm, and spleen in athymic and non-obese diabetic/severe combined immunodeficient mice (NOD/SCID) following injection into the prostate [134]. GPR4 over-expression reduced lung metastasis of B16F10 melanoma cells in C57BL/6 mice [133]. GPR4 has also been implicated in the alteration of cell metabolism in B16F10 melanoma cells [135]. In a study of B16F10 melanoma cells that over-express GPR4, the maximal oxygen consumption rate (OCR) of mitochondria was increased [135]. In addition, the mitochondrial surface area and volume were increased as well [135]. GPR65 has been implicated as a potential tumor suppressor in hematological malignancies [124]. According to the Oncomine database and a recent publication, GPR65 expression is reduced in lymphoma and leukemia samples when compared to normal lymphoid tissues [124]. Of the proton sensing G-protein coupled receptors GPR65 expression and activity in particular may alter cancer cell metabolism in response to acidosis [124]. Acidosis activation of GPR65 has been reported to reduce c-Myc expression in lymphoma and leukemia cells [124]. This may indicate that the development of an agonist of GPR65 to reduce c-Myc expression may be beneficial for future therapeutic applications. GPR65 has also been shown to enhance corticosteroids-induced lymphoma cell apoptosis [136]. In contrast, GPR65 has been found to increase the cell survival response to acidosis in murine T-cell lymphoma cells (WEHI7.2) and human acute lymphoblastic leukemia cells (CEM-C7) under glutamine starvation conditions [137]. GPR65 response to acidosis was also investigated in Lewis lung carcinoma cells (LLC) [138]. Its over-expression was found to increase cell survival under acidic conditions by activating protein kinase A (PKA) and the extracellular signal related kinase (ERK) pathways [138]. In addition, the over-expression of GPR65 in LLC cells was found to cause the up-regulation of genes that facilitate glycolytic activity [138]. The response of this family of receptors is complex and cell type dependent but learning how they function may increase our understanding of acidosis effects on tumor cells in the tumor microenvironment.

## 6. Implications for Cancer Therapy and Chemoprevention

### 6.1. Metabolic Pathways and Cancer Therapy

Exploiting the characteristic cancer cell metabolism by targeting specific metabolic pathways is an attractive stratagem for cancer therapy. Some pre-clinical studies have targeted GLUT transporters as a means to derail glycolytic conversions and sequester glucose from cancer cells [139,140]. Targeting glucose uptake, however, may have adverse side effects on normal cells, which express GLUT transporters and rely on glucose for normal cellular metabolism. Looking beyond glucose uptake, the first rate-limiting step of glycolysis is a potential target for cancer therapy.

There are a number of chemical inhibitors designed to reduce glycolytic flux governing the rate limiting step of hexokinase (HK) activity. One such therapeutic is a glucose analog 2-deoxy-d-glucose (2-DG), which is a competitive inhibitor for normal glucose metabolism [141]. Once transported into the cell, 2-DG is phosphorylated by HK. When phosphorylation of 2-DG occurs, yielding 2-deoxyglucose-6-phosphate (2-DG-P), glucose-6-phosphate isomerase cannot further metabolize 2-DG-P. As 2-DG-P accumulates in the cell, 2-DG-P competitively inhibits HK. This phosphorylation event, followed by 2-DG-P accumulation, causes a depletion of ATP levels, a reduction in cell cycle progression, and apoptosis. [142,143,144]. In addition, utilizing glucose analogs such as 2-DG has proven beneficial in cancer diagnosis. ^18^Fluoro-deoxyglucose (FDG) is an analog of 2-DG that has been used clinically as a diagnostic tool for measuring tumor progression and therapeutic response using positron emission tomography (PET) [145,146]. Pre-clinical studies presented 2-DG as possessing strong anticancer effects, however, there have been several demonstrations indicating some limitations in using 2-DG in regard to patient tolerability [147]. Some of the most common side effects of 2-DG are similar to that of hypoglycemia [147]. One study suggested effective dosing of 2-DG cannot balance with patient tolerability of the agent. Patients have acceptable toxicity when treated with 2-DG at low doses (200–250 mg/kg body weight), however, these lower doses may not be sufficient in effectively inhibiting glycolysis of cancer cells [148]. Doses that are effective in therapy are reported at 300 mg/kg body weight, but this dose was too strenuous on patients and they were unable to complete the treatment. These limitations may be overcome if 2-DG is used in combination with other anticancer agents [149]. One study suggests that 2-DG in combination with cisplatin has synergistic effects by increasing metabolic oxidative stress and also results in substantial tumor cell cytotoxicity in head and neck cancers [150,151]. In addition to 2-DG, there are other drugs that inhibit Hexokinase I/II. Some such drugs are 3-bromopyruvate and Lonidamine [152,153]. 3-bromopyruvate, another glycolytic inhibitor, has shown promise in selective cytotoxicity to cancer cells but has only recently progressed passed pre-clinical studies into clinical trials [154]. Lonidamine showed benefits in inhibiting glycolysis in cancer cells while increasing glycolysis in nontransformed cells. These pre-clinical studies evidenced Lonidamine as possessing merit as a chemotherapeutic [155,156,157]. Lonidamine reached phase III of clinical trials [158,159,160], however, there has not been further advancement with this agent recently. Increased toxicity to the pancreas and liver by Lonidamine has been reported in an animal model [161].

In addition to inhibiting HK2, glucose 6-phosphate dehydrogenase (G6PD) is an enzyme to target as it facilitates the glycolytic shunt into the pentose phosphate pathway. 6-aminonicotinamide (6-AN) is a chemical inhibitor for G6PD and has shown promise during *in vitro* pre-clinical studies, however, there have been neurotoxic effects recorded by 6-AN [162,163]. Combination therapy of 6-AN with 2-DG sensitizes the cancer cells to radiation therapy by inhibiting the PPP and thereby reducing glutathione levels to eliminate ROS generation [164]. DCA is another glycolytic inhibitor. DCA targets PDK1 and thereby shifts metabolism away from lactic acid production and towards mitochondrial oxidative phosphorylation [165]. A preliminary clinical trial demonstrated DCA as an effective means in treating glioblastoma [166]. Another study with a human cancer patient, diagnosed with non-Hodgkin’s lymphoma, received DCA monotherapy after relapse from treatment with rituximab-CHOP [167]. This patient achieved complete remission [167]. These results warrant further investigation of DCA in clinical studies.

Beyond glycolysis, targeting the TCA cycle is yet another potential direction for cancer therapy. Metformin, a clinically approved drug by the FDA to treat type II diabetes, targets the mitochondrial complex I and thereby reducing ATP synthesis [168,169]. Interest in metformin as a cancer therapeutic was sparked when patients with diabetes showed a reduced risk of cancer related death when taking metformin [170,171,172]. This observation lead to many clinical trials evaluating metformin as a cancer preventative agent [173]. Currently, in early clinical trials, metformin is also being evaluated as a cancer prevention and treatment option in a variety of cancer types [173]. There is controversy over the exact mechanism of the proposed anti-cancer effects of metformin, however, it is generally accepted that metformin activates AMPK when AMP/ATP ratios increase [169]. One study suggests the selectivity of metformin against cancer cells is due to the activation of AMPK and subsequent inhibition of mTOR signaling to downstream effectors such as S6K1 and 4EBP1 [174]. Signaling through mTOR is critical for cancer cell survival and proliferation [174]. Arsenic trioxide (ATO), a mitochondrial toxicant, is currently used in the treatment of acute promyelocytic leukemia (APL) [175,176]. ATO has several mechanisms by which APL is targeted. One such mechanism is by inducing apoptosis and partial myeloid differentiation by collapsing the mitochondrial transmembrane potential [177]. Another mechanism by which ATO targets APL is through directly binding and degrading the APL specific oncoprotein PML-RARα [178]. ATO is the principle agent in APL treatment despite some toxicity risks associated with treatment. Mutations of isocitrate dehydrogenase (IDH), another component of the TCA cycle, are frequently found in several types of cancer such as glioma and acute myeloid leukemia [179,180]. Inhibitors of IDH mutants have been developed and demonstrated anti-cancer activities [181,182].

mTOR, which is downstream of PI3K/AKT and governs many metabolic responses to energy availability, is currently targeted with various rapalogs in the treatment of breast cancer, pancreatic neuroendocrine tumor, acute leukemia, and renal cell carcinoma [183,184,185,186,187,188,189,190,191,192,193]. Rapalogs bind to peptidyl-prolyl *cis*-*trans* isomerase, FKBP12, to inhibit activity of the mTORC-1 complex thereby inhibiting mTOR’s ability to stimulate cell growth, angiogenesis, and nutrient transport [194]. Several rapalogs such as temsirolimus (Torisel) [183,185] and everolimus (Affinitor) [184,191] have been FDA approved and are effective in treatment of renal cell carcinoma. Everolimus (Affinitor), is also FDA approved for the treatment of pancreatic neuroendocrine tumors [192,193], pediatric and adult subependymal giant cell astronoma [188,189], and advanced hormone receptor positive HER-2 positive breast cancer in post-menopausal women [186,187]. Although rapalogs may work to inhibit mTOR activity, PI3K/AKT up-regulation following treatment is of concern. The use of rapalogs in combination with PI3K and AKT inhibitors are being actively evaluated to inhibit this response [190]. In summary, targeting the metabolic pathways of tumor cells with new therapeutic options has great potential for cancer treatment but understanding how metabolic signaling occurs is crucial before their use.

### 6.2. Hypoxia and Cancer Therapy

This section will review the molecular signaling pathways that govern the hypoxic response and how these pathways may be targeted as a means for cancer therapy. As discussed previously, HIF-1 is the main regulator of the hypoxic response. HIF-1 facilitates the adaption and selection of tumor cells for survival under hypoxic stress. In addition, HIF-1 activation directly alters tumor cell metabolism and facilitates angiogenesis to increase blood supply and tumor growth. Great effort has gone into directly targeting HIF-1 or its target genes of which regulate metabolism and angiogenesis [195,196,197]. Regulation of HIF-1α for therapy can be aimed in several directions. Some such approaches are regulating HIF-1α by inhibiting DNA binding, HIF-1 translation, and HIF-1α degradation [195,196,197]. Flavopiridol is a chemical agent that inhibits *HIF-1α* gene transcription and is in clinical trials [198,199]. Some chemicals such as Acriflavine and Digoxin have been developed to inhibit HIF-1 dimerization and synthesis, thereby blocking tumor growth and vascularization [200,201]. Furthermore, P276-00, a cyclin-dependent kinase inhibitor, has been shown to inhibit HIF-1α transcription and induce G2/M cell cycle arrest in prostate cancer cells under hypoxic conditions [202,203]. Finally, trichostatin A (TSA) has proven to degrade HIF-1α by inhibiting histone deacetylases [204].

The HIF target genes *VEGF* and *VEGFR* are critical for angiogenesis and are therefore an ideal target for therapy by eliminating the much needed supply of oxygen and nutrients to the tumor. Additionally, anti-angiogenesis therapy would potentially increase chemotherapy effectiveness by reducing abnormal blood vessel growth and increasing the absorbency of the tumor to chemotherapeutics. VEGF blockade is currently the most predominate strategy in anti-angiogenesis therapy. Targeting strategies for VEGF are use of anti-VEGF antibodies and VEGFR tyrosine kinase inhibitors. Bevacizumab is a monoclonal antibody that specifically targets VEGF and is commonly used clinically for anti-angiogenesis therapy [205,206,207]. Sorafenib, sunitinib, and pazopanib are multi-targeted kinase inhibitors that have been approved by the FDA and are used to target multiple cancer types [208,209,210,211]. However, targeting VEGF for anti-angiogenesis therapy has proven to be limited in its effectiveness in some cases as there are multiple mechanisms by which tumors can lose sensitivity to anti-VEGF therapy. The benefits of targeting VEGF and VEGFR can be short lived as the tumor can quickly recover vascular growth through other compensatory pro-angiogenic signaling pathways [212,213,214]. Nonetheless, targeting VEGF and VEGFR for anti-angiogenesis therapy is a growing field with room for much advancement. Another target for anti-angiogenesis therapy is the mTOR pathway. As many cancer cells are dependent on the previously described PI3K-Akt-mTOR pathway for growth and survival, so too are endothelial cells [215]. Under a hypoxic condition, mTOR may signal to increase VEGF expression to stimulate endothelial cell proliferation. Inhibition of mTOR signaling by rapamycin can reduce VEGF expression and suppress angiogenesis [215].

### 6.3. Acidosis and Cancer Therapy

Throughout the development and growth of a solid tumor, as discussed previously, the tumor microenvironment may become acidic for multiple reasons. There are numerous reports that indicate acidosis modifies the tumor cell response to chemotherapeutical intervention. For example, acidosis in the tumor microenvironment may reduce the cytotoxicity of certain chemotherapeutical drugs such as cisplatin, teniposide, vincristine, and topotecan, among others [216,217,218,219]. An additional problem that exists is the ability of acidosis to alter drug uptake. The uptake of weakly basic drugs in tumors with low pH is reportedly reduced whereas weakly acidic drugs were taken up more readily [216,219]. This phenomenon should be taken into account before administration of chemotherapeutics. As acidosis in the tumor microenvironment may influence the uptake and effectiveness of certain drugs, some studies suggest that acidosis may be used to its advantage [220,221,222]. There are several different types of nanoparticles being developed that are activated by acidic pH [220,221,222]. These nanoparticles, such as low pH insertion peptides (pHLIP) and polyethylene glycol (PEG) based hydrogels integrated with imidazole, are engineered to release chemotherapy drugs, imaging agents or genetic constructs in response to acidosis [220,221,222]. This may reduce the non-specific or systemic adverse effects commonly associated with chemotherapy. These new developments may also be advantageous for imaging purposes and may be applicable to solid tumors, ischemia, stroke, and infection [220,221,222]. Furthermore, to reduce the effects of acidosis on drug activity under acidic conditions, systemic buffer therapy has been proven to be effective in alkalizing the tumor microenvironment [223,224,225]. In several reports oral administration of buffers such as lysine, sodium bicarbonate, or 2-imidazole-1-yl-3-ethoxycarbonylpropionic acid (IEPA) was used to systemically buffer mice in order to reduce tumor growth and metastasis [223,224,225]. Using systemic buffers may increase the activity of certain drugs that are normally inactive in such acidic environments [216,219]. Recently, nanoparticles that target both hypoxia and acidosis of the tumor microenvironment have been developed [226]. These bioinorganic nanoparticles are composed of polyelectrolyte-albumin complex and MnO_2_ that rescue the oxygen depleted environment by the reactivity of MnO_2_ with ROS, such as H_2_O_2_, and increasing the tumor pH from 6.7 to 7.2. This therapy has been shown to reduce tumor growth, down-regulate HIF-1, VEGF, and increase cancer cell death when combined with radiation therapy in a mouse model [226]. Exciting new cancer therapies such as these are on the horizon in the direct targeting of the tumor microenvironment.

There are a few other strategies for manipulating tumor pH as a means for therapy. Proton pump inhibition is an area of investigation for using acid-base transporters as a means to increase the intracellular acidity and overcome the reversed pH gradient associated with cancer cells. Proton extrusion is accomplished by many transporters in cancer. Among the most prominent are vacuolar H^+^-ATPase, carbonic anhydrases, Na^+^/H^+^ exchangers, and monocarboxylate transporters. Inhibiting these mechanisms in the tumor cells by chemical inhibitors may stimulate a pH crisis for tumor cells and lead to cell death. Bafilomycin A1, a V-ATPase proton pump inhibitor, was used to treat human hepatoblastoma cells [227]. The results showed selective toxicity to hepatoblastoma cells and non-caustic to normal hepatocytes [227]. Hepatoblastoma cell growth was reduced while apoptosis was induced after Bafilomycin A1 treatment [227]. In addition, the inhibition of vacuolar H^+^-ATPase with archazolid, a known inhibitor of V-ATPase, was cytotoxic to SKBR3, MDA-MD-231, 4T1-Luc2 breast adenocarcinoma cells [228]. Another study investigated the therapeutic effects using proton pump inhibitors, such as omeprazole, esomeprazole, rabeprazole, pantoprazole, and lansoprazole, for the suppression of acid production associated with increased risk of developing Barrett’s esophagus (BE) related esophageal adenocarcinoma [229]. The results indicated patients with BE, over 5.2 years, had a reduced risk of neoplastic progression given proton pump inhibitors [229]. It was unknown whether this was an indirect effect of lessening the damage to the esophagus over time or if it was a direct effect on cancer cell transformation. The direct effect of this class of drugs on cancer cells were, however, published in several other reports. Omeprazole reportedly prevented azoxymethane-induced colonic aberrant crypt foci development in male F344 rats as well as reducing HCT-116 and HCA-7 colon cancer cell growth [230]. Esomeprazole was found to inhibit MDA-MB 468 triple negative breast cancer cell growth while rabeprazole had growth inhibitory effects on MKN 28 and AGS gastric cancer cell lines [231,232]. In addition, lansoprazole has been investigated for its ability to stimulate MDA-MB-231 breast cancer cell death in a dose dependent manner [233]. Treatment of nude mice with lansoprazole also had significant inhibitory effects on MDA-MB-231 tumor xenograft growth [233]. Pantoprazole has been found to enhance activity of docetaxel against human tumor xenografts by inhibiting autophagy [234]. Furthermore, pantoprazole is also in clinical trials for treatment of advanced solid tumors in combination with doxorubicin [235]. These studies suggest that inhibitors of proton transporters may have potential for cancer prevention or treatment. The ability of acidosis to alter tumor cell metabolism through the activation of proton sensing G-protein coupled receptors may be beneficial for the development of future treatment options. The capability of proton sensing G-protein coupled receptors to alter tumor cell metabolism is discussed previously in this review [14,124,135]. As more than 30% of all currently utilized drugs (e.g., agonists, antagonists, and antibodies, among others) on the market today target G-protein coupled receptors for the treatment of human diseases, the development of therapeutic agents directed toward this family of receptors may be a valid option for cancer treatment or prevention [236]. Understanding how the activation of these receptors may alter tumor cell metabolism through complex molecular signaling may permit us to activate or inhibit pathways with small molecules to inhibit tumor cell proliferation. In this regard, an agonist of GPR68 has been shown to inhibit malignant astrocyte proliferation [237]. Furthermore, as novel delivery systems become available [220,221,222], the precision at which these new small molecules may be introduced into the tumor without affecting normal cells will become more defined.

## 7. Conclusions

The classical observation of increased glycolysis in cancer cells when compared to their normal counterparts initiated great research interest in cancer cell metabolism. Now altered cancer cell metabolism is perceived as a hallmark of cancer and is extensively examined. A key connection was identified linking oncogenes and tumor suppressors with deregulated cancer cell metabolism. This connection sheds light into how intrinsic cellular factors can influence cancer cell metabolism and modulate the tumor microenvironment that has the characteristic features of hypoxia and acidosis. The extrinsic factors of the tumor microenvironment, particularly hypoxia and acidosis, can reciprocally regulate the intrinsic signaling of oncoproteins and tumor suppressors as well as cancer cell metabolism. The information gained has helped define new and innovative treatment options that are being used currently and will be used in the future. Further understanding of the complex and reciprocal nature of cancer cells within their microenvironment is needed for a more comprehensive view of tumor biology.
